# *Panax ginseng*-Derived Extracellular Vesicles Facilitate Anti-Senescence Effects in Human Skin Cells: An Eco-Friendly and Sustainable Way to Use Ginseng Substances

**DOI:** 10.3390/cells10030486

**Published:** 2021-02-24

**Authors:** Eun-Gyung Cho, Suh-Yeon Choi, Hyoseon Kim, Eun-Jeong Choi, Eun-Jeong Lee, Phil-Jun Park, Jaeyoung Ko, Kwang Pyo Kim, Heung Soo Baek

**Affiliations:** 1 Basic Research and Innovation Division, R&D Center, Amorepacific Corporation, Yongin 17074, Korea; sychoi@amorepacific.com (S.-Y.C.); ejchoi@amorepacific.com (E.-J.C.); ejlee80@amorepacific.com (E.-J.L.); mosme@amorepacific.com (P.-J.P.); jaeyoungko@amorepacific.com (J.K.); monmimi@amorepacific.com (H.S.B.); 2 Department of Applied Chemistry, College of Applied Science, Kyung Hee University, Yongin 17104, Korea; invaluably@naver.com (H.K.); kimkp@khu.ac.kr (K.P.K.)

**Keywords:** *Panax ginseng* C.A. Meyer, extracellular vesicle, ginseng cell, human skin cell, anti-senescence, anti-pigmentation, lipidomic analysis, natural nanomaterial

## Abstract

Ginseng is a traditional herbal medicine in eastern Asian countries. Most active constituents in ginseng are prepared via fermentation or organic acid pretreatment. Extracellular vesicles (EVs) are released by most organisms from prokaryotes to eukaryotes and play central roles in intra- and inter-species communications. Plants produce EVs upon exposure to microbes; however, their direct functions and utility for human health are barely known, except for being proposed as delivery vehicles. In this study, we isolated EVs from ginseng roots (GrEVs) or the culture supernatants of ginseng cells (GcEVs) derived from *Panax ginseng* C.A. Meyer and investigated their biological effects on human skin cells. GrEV or GcEV treatments improved the replicative senescent or senescence-associated pigmented phenotypes of human dermal fibroblasts or ultraviolet B radiation-treated human melanocytes, respectively, by downregulating senescence-associated molecules and/or melanogenesis-related proteins. Based on comprehensive lipidomic analysis using liquid chromatography mass spectrometry, the lipidomic profile of GrEVs differed from that of the parental root extracts, showing significant increases in 70 of 188 identified lipid species and prominent increases in diacylglycerols, some phospholipids (phosphatidylcholine, phosphatidylethanolamine, lysophosphatidylcholine), and sphingomyelin, revealing their unique vesicular properties. Therefore, our results imply that GEVs represent a novel type of bioactive and sustainable nanomaterials that can be applied to human tissues for improving tissue conditions and targeted delivery of active constituents.

## 1. Introduction

Extracellular vesicles (EVs) are lipid bilayer-enclosed spheres that are released by all living cells (ranging from prokaryotic to eukaryotic cells) into the extracellular environment, and this process is developmentally conserved from bacteria to humans and plants [1]. EVs appear to take charge of cell-to-cell communications by transferring proteins, nucleic acids, and lipids locally, at a distance, or even across kingdoms, thereby regulating many pathophysiological situations, including cancer, immune responses, regeneration, and host–pathogen interactions [2]. Although EVs are heterogeneous in terms of their origins, sizes, and molecular compositions, they are classified into two major populations according to their biogenesis [2,3,4]. These populations include (i) exosomes (30–100 nm in diameter) formed by inward budding of endosomal membranes during the maturation of multivesicular bodies (MVBs) and secreted upon MVB fusion with the plasma membrane and (ii) microvesicles (50–1000 nm in diameter) generated by the outward budding and fission of the plasma membrane.

In contrast to EVs derived from mammalian or bacterial cells (whose secretion, uptake, and functions in inter-cellular communications, physical characteristics, and vesicular components have been widely verified [1,5,6,7,8,9,10,11,12], the corresponding properties of plant-derived EVs are barely known, although EVs were discovered in plants before they were identified in mammals [13]. Since exosome-like vesicles (EVs henceforth) were first isolated from apoplastic fluids of water-imbibed sunflower seeds using a standard differential ultracentrifugation method [14], EVs have been isolated from several plants, including ginger [15,16], grapefruits [17], grapes [18], coconut water [19], *Arabidopsis* leaves [20,21], broccoli [22], and *Citrus limon* L. [23]. In addition, EVs have been studied in the contexts of non-classical protein secretions and cell wall remodeling [24,25], microbial interactions important for immunity or defense [26,27,28], stress responses [20], and as nanocarriers for compounds or bioactive substances such as small interfering RNAs (siRNAs) and microRNAs (miRNAs) [15,29,30,31]. Numerous reports have described the production of synthetic exosome-like nanovectors, which can be produced from serial extrusions of cells or by bottom-up synthesis. Both natural plant-derived EVs and mimic nanovectors are currently considered good alternatives for drug delivery because they are easier to extract, do not have the drawbacks of those produced in animal cells, and have shown therapeutic effects against bowel diseases, colitis, tumors, alcohol-induced liver damage, and gut microbiota in established mice models [15,16,17,18,22,31,32,33,34]. Nonetheless, except for the anti-oxidant activity of *Citrus limon* L.-derived EVs [23], little is known about the biological effects of plant-derived EVs, particularly in human tissues.

The Korean ginseng (*Panax ginseng* C.A. Meyer) root has been used as a traditional herbal medicine for over 2000 years in east Asian countries due to its various beneficial effects on human health, e.g., immunomodulatory, heart protective, anti-cancer, and neuroprotective properties [35,36,37]. Although various active constituents, including polysaccharides, peptides, polyacetylenic alcohols, and fatty acids, are also expected to exert pharmacological effects [38], most pharmacological actions of ginseng have been attributed to ginsenosides (over 50 different kinds) [39,40], which have been the focus of large studies. These ginsenosides, however, need to be metabolized and transformed by intestinal microbes before being absorbed in the human intestines and for efficacy in human tissues, necessitating the application of fermentation or organic acid pretreatment strategies [41]. Bioactive constituents besides ginsenosides, e.g., peptides/proteins, nucleic acids, and lipids, including metabolites, are also predicted to be effective in human health, although their effects, mechanisms of action, and acquisition methods have been barely evaluated.

Here, we found that ginseng-derived EVs (GEVs) could be purified from the extracts of ginseng roots or the supernatants of cultured ginseng cells (which are generally discarded without finding a use for them) using a standard differential ultracentrifugation method. The GEVs exhibited morphologies and sizes typical of EVs and showed anti-senescence and anti-pigmentation effects in human skin cells. Through lipidomic analysis, we found that some lipid species were detected only in GEVs. In particular, diacylglycerol and several phospholipid classes were significantly enriched in GEVs compared to their parent root extracts, suggesting that the GEVs possessed a unique vesicular characteristic and can potentially be used as alternative and sustainable bioactive substances and delivery vehicles.

## 2. Materials and Methods

### 2.1. Purification of GEVs

To purify EVs from ginseng roots (GrEVs), fresh ginseng roots (about 500–750 g) were collected from 5–10 plants of 4 year old Korean ginseng (*P. ginseng* C A Meyer) that were grown in the open field and purchased through the farm, and washed multiple times with water. The ginseng roots were ground, and ginseng root extracts (GrEXs) were prepared using a Hurom slow juicer (Gimhae, South Korea). GrEXs were sequentially centrifuged at 500× *g* for 10 min and at 3000× *g* for 20 min to remove cell debris, fibers, and large particles. The clear juice (34 mL/tube) was loaded on the top of a sucrose cushion with two layers (1 mL of 0.8 M and 0.5 mL of 2 M sucrose) and ultracentrifuged at 100,000× *g* for 2 h (SW 32 Ti rotor, Beckman Coulter, Brea, CA, USA). Following ultracentrifugation, the GrEV fraction (located between both layers of the sucrose cushion) was collected and assessed in terms of its biological effects in human skin primary cells. To purify GcEVs, the culture supernatants of ginseng cells were collected at 2 weeks post-cultivation in bioreactors. The supernatants were sequentially centrifuged at 500× *g* for 10 min and at 3000× *g* for 20 min to remove cellular debris. The resulting supernatants were ultracentrifuged at 100,000× *g* for 3 h, and the pellets were dissolved in 4-(2-hydroxyethyl)-1-piperazineethanesulfonic acid (HEPES)-buffered saline (HBS).

To perform cryo-electron microscopy (cryo-EM) analysis and validate the biological effects of GrEVs, GrEVs were further purified via density gradient ultracentrifugation using OptiPrep solution (Axis-Shield PoC AS, Oslo, Norway) according to the manufacturer’s protocol with specific modifications and following previously described methods [5]. Briefly, after sucrose-cushion ultracentrifugation, the GEV fractions were mixed with five volumes of OptiPrep solution, composed of a 60% (*w*/*v*) solution of iodixanol in water. Next, 2.5 mL of control solution (50% iodixanol alone) or GEVs in 50% iodixanol was overlaid with 2.5 mL each of 35%, 20%, and 0% iodixanol solutions, which were prepared by mixing the OptiPrep solution with a homogenization medium (0.25 M sucrose, 150 mM NaCl, 20 mM HEPES, pH 7.4), and then centrifuged at 200,000× *g* at 4 °C for 2 h (SW 41 Ti rotor, Beckman Coulter). One milliliter fractions were collected from the top of the gradient of the control tube, and the density of each fraction was determined using a standard curve based on absorbance values at 340 nm. The EV fraction with a density of approximately 1.08 (fraction 3) was collected from the sample tube using a 1 mL syringe. To remove the iodixanol, the fraction was diluted in 60 mL of HBS, ultracentrifuged at 150,000× *g* at 4 °C for 2 h, and resuspended in 200 μL of HBS. The total protein concentration was determined by performing a Bradford assay (Bio-Rad Laboratories, Hercules, CA, USA). Distribution, average diameter, and particle numbers of GrEVs, GcEVs, and density-purified GrEVs were measured by dynamic light scattering (DLS) using a Zetasizer Nano ZS instrument (Malvern Instruments, Worcestershire, UK) or by tunable resistive-pulse sensing (TRPS) using a qNano Gold-equipped NP80 nanopore (Izon Science, Christchurch, New Zealand). The purified GEVs were stored at −80 °C until use.

### 2.2. Cryo-EM Analysis

Cryo-EM images were obtained as previously described [8]. Briefly, 3 µL of GrEVs (purified by density gradient ultrapurification with OptiPrep solution) was added to both sides of a Quantifoil TEM grid with a 1.2 μm diameter hole and an inter-hole distance of 1.3 μm. The TEM grid was blotted for 1.5 s and plunged into liquid ethane using a Cryoplunge 2 system (Gatan, Pleasanton, CA, USA). The cryo-EM samples were stored in liquid nitrogen before performing the TEM observations. The samples were examined under a Tecnai F20 electron microscope operated at 120 kV (Hillsboro, OR, USA).

### 2.3. Cell Culture

Neonatal human epidermal keratinocytes (HEKs; Lonza, Basel, Switzerland) were cultured in KBM-Gold medium supplemented with the components of the KGM-Gold Bullet Kit (Lonza) for 4 or 12 days, during which time normal growth and spontaneous terminal differentiation occurred. HEKs were subjected to ultraviolet B (UVB) irradiation (30 mJ/cm^2^) using a Bio-Sun irradiation system (Vilber Lourmat, Marne-la-Vallée, France FranceFrance) and then cultured for 4 days. Human dermal fibroblasts (HDFs) derived from neonatal foreskin (Lonza) were cultured in Dulbecco’s modified Eagle’s medium supplemented with 10% fetal bovine serum (FBS) (Lonza). Human epidermal melanocytes (HEMs) derived from neonatal foreskin of moderately pigmented donors (Cascade Biologics, Portland, OR, USA) were cultured in Medium 254 supplemented with human melanocyte growth supplement (HMGS; Life Technologies, Carlsbad, CA, USA). All culture media contained 100 unit/mL of penicillin-streptomycin (Thermo Fisher Scientific, Waltham, MA, USA), and all cells were cultured at 37 °C in the presence of 5% CO_2_.

### 2.4. Induction of Cellular Senescence

Replicative senescence in HDFs was induced by transferring confluent cells to two new culture dishes, expanding them to confluence, and doubling the population, as previously reported [9]. Cellular senescence and senescence-associated (SA) pigmentation in HEMs were induced as previously reported [42]. Briefly, 24 h after plating, the supernatant was replaced with phosphate-buffered saline (PBS) with calcium and magnesium (Corning Life Science, Corning, NY, USA) and irradiated twice with UVB (20 mJ/cm^2^) using a Bio-Sun irradiation system (Vilber Lourmat) over a 24 h period and subsequently cultivated for 2 weeks in Medium 254 supplemented with HMGS. Non-irradiated control cells were cultured under the same conditions.

### 2.5. Cell-Proliferation Assay

Cell proliferation was measured using the WST-1 cell proliferation reagent (Roche Applied Science; Penzberg, Upper Bavaria, Germany). Briefly, 7 × 10^3^ HEM cells were placed in each well of 12 well plates and treated with different concentrations of GrEVs. Cell viability was tested after 48 h, according to the manufacturer’s instructions.

### 2.6. SA β-Gal Assay

The SA β-gal activities of senescent HDFs or HEMs were determined using a Mammalian β-Gal Assay Kit (Thermo Fisher Scientific), according to the manufacturer’s instructions. HDFs or HEMs (2 × 10^5^), either non-treated or treated with GrEVs or GcEVs, were collected in PBS, and proteins were extracted using the M-PER mammalian protein extraction reagent (Thermo Fisher Scientific). After centrifugation at 3000× *g* for 15 min, 50 μL β-Gal assay reagent was added to 50 μL supernatant. The reaction proceeded for 30 min at 37 °C, and absorbance was measured at 405 nm with a Synergy H2 microplate reader (BioTek, Winooski, VT, USA).

### 2.7. Reverse Transcription-Quantitative Polymerase Chain Reaction (RT-qPCR) Analysis

Total RNA was extracted from cells using the TRIzol^®^ reagent (Thermo Fisher Scientific) and quantified using a NanoDrop One spectrophotometer (Thermo Fisher Scientific). RNA (100 ng) was reverse transcribed using a RevertAid RT Kit (Thermo Fisher Scientific), following the manufacturer’s instructions. qPCR was performed using a 7500 Fast Real-Time PCR system (Applied biosystems; Thermo Fisher Scientific) and TaqMan™ Gene Expression Master Mix (Thermo Fisher Scientific), per the manufacturer’s instructions. Thermal cycling was performed as follows: 5 min at 95 °C followed by 40 cycles of 15 s at 95 °C and 60 s at 60 °C. Primers and hydrolysis probe sets (TaqMan Gene Expression Assays) for tumor protein p53 (*TP53*), p21^Cip1^ (*CDKN1A*), p16^INK4a^ (*CDKN2A*), matrix metallopeptidase 1 (*MMP1*), interleukin 8 (*IL8*), keratin 10 (*KRT10*), loricrin (*LOR*), and filaggrin (*FLG*) were purchased from Thermo Fisher Scientific. Gene expression levels were determined using the 2^−ΔΔCq^ method, and all data were normalized based on ribosomal protein L13a (*RPL13A*) expression. Three independent treatments using two independent senescent cell lines from one donor were performed in HDFs, and three independent treatments were performed in HEKs.

### 2.8. Melanin Assay

HEMs were lysed by sonication in radioimmunoprecipitation assay (RIPA) buffer (0.1 M Tris-HCl (pH 7.2) containing 1% Nonidet P-40, 0.01% sodium dodecyl sulfate, and a protease inhibitor cocktail; Roche Applied Science) and centrifuged at 15,000× *g* for 10 min. Protein concentrations in culture supernatants were quantified using a BCA Protein Assay Kit (Pierce; Thermo Fisher Scientific). Pellets containing melanin were dissolved in 1 N NaOH and incubated for 30 min at 60 °C. Protein concentrations and melanin contents were determined by measuring the absorbance at 562 nm and 450 nm, respectively, using a Synergy H2 microplate reader (BioTek). The melanin contents were normalized to the protein concentrations.

### 2.9. Western Blot Analysis

Cells (2 × 10^6^) were lysed in RIPA buffer (Sigma-Aldrich Corp., St. Louis, MO, USA) containing protease and phosphatase inhibitor cocktail (Sigma-Aldrich Corp.) for 30 min at 4 °C, followed by centrifugation at 3000× *g* for 30 min. Protein concentrations were determined using a BCA Protein Assay kit (Thermo Fisher Scientific), and 25 µg of total protein was loaded onto a 5–20% gradient gel. Subsequently, immunological analyses were performed using antibodies against p53 (Cell Signaling Technology, Danvers, MA, USA), tyrosinase (TYR) (Upstate; Thermo Fisher Scientific), TYRP1, TYRP2, glyceraldehyde 3-phosphate dehydrogenase (GAPDH; Santa Cruz Biotechnology, Dallas, TX, USA), Ras-related protein 27 (RAB27), and high-mobility group box 1 (HMGB1) (Abcam, Cambridge, UK). Densitometry analysis of each band intensity was performed using ImageJ software (https://imagej.nih.gov/ij/).

### 2.10. Ginseng Cell Induction and Suspension Culture

Calli were induced from the root segments (1–2 cm) of 5 year old Korean ginseng plants as described previously [43,44,45] with some modifications. Briefly, the root segments were incubated on semi-solid Marashige and Skoog medium (Sigma-Aldrich Corp.) supplemented with 1 mg/L 2,4-D (Duchefa Biochemie, Haarlem, Netherlands), 3% sucrose, and 2.3 g/L Gelrite (Duchefa Biochemie) in the dark at 20 °C. Actively growing ginseng cells, which migrated out from each callus, were selected and cultured for 2 weeks in liquid Marashige and Skoog medium supplemented with 1 mg/L 2,4-D and 3% sucrose in 3 L, bulb-type bioreactors containing 2 L medium. The FWs of cultured ginseng cells were measured, and the cells were imaged under an optical microscope (Olympus IX73; Tokyo, Japan). The culture supernatants were collected by sequential centrifugation at 500× *g* for 10 min and at 3000× *g* for 20 min. The supernatants were filtered through 0.45 µm membrane filters and then subjected to ultracentrifugation for GcEV purification.

### 2.11. Lipid Standards

The following lipid standards used in this study were purchased from Larodan Fine Chemicals AB:TG (11:1-11:1-11:1), diacylglycerol (DG) (8:0-8:0), monoacylglycerol (MG) (15:1), and cholesteryl ester (CE) (10:0). Additional lipid standards were purchased from Avanti Polar Lipids, Inc., including phophatidylcholine (PC) (10:0-10:0), phosphatidylethanolamine (PE) (10:0-10:0), phosphoserine (PS) (10:0-10:0), phosphatidylglycerol (10:0-10:0), phosphatidylinositol (8:0-8:0), phosphatidic acid (PA) (10:0-10:0), lysophosphatidylcholine (LPC) (13:0), lysophosphatidylethanolamine (LPE) (14:0), lysophosphoserine (LPS) (17:1), lysophosphatidylglycerol (14:0), lysophosphatidylinositol (13:0), lysophosphatidic acid (LPA) (14:0), sphingomyeline (SM) (d18:1-12:0), and ceramide (Cer) (d18:1-12:0). Each lipid standard was dissolved in methanol or chloroform (in the case of CE), stored at −20 °C, and diluted to 1 µg/mL for extraction.

### 2.12. Lipid Extraction

A two-step extraction method was performed for high efficiency extraction of polar and non-polar lipids from GrEXs and GrEVs according to previously reported methods [7]. First, samples were lysed by adding 5 μL of 0.1% sodium dodecyl sulfate (SDS) in PBS into 45 μL of each sample in PBS (final 0.01% SDS in PBS) and incubating for 20 min at 37 °C. After sonication (program: pulse on for 5 s, pulse off for 10 s, 10% amplitude) for 3 min on ice using a probe sonicator (Branson, Danbury, CT, USA), the lysed samples were centrifuged at 13,800× *g*, 20 min at 4 °C. The supernatants were collected, and protein concentration was determined using a BCA Protein Assay kit (Thermo Fisher Scientific). Samples set to contain the same amount of 30 μg protein in 50 μL of 0.01% SDS in PBS were added to 990 µL methanol/chloroform (2:1, *v*/*v*; 660 μL:330 μL) with 10 μL of internal standard (IS) lipids (1 µg/mL; a mixture of IS lipids). Each sample was vortexed three times (30 s each) and incubated for 10 min on ice. After centrifugation (13,800× *g*, 2 min at 4 °C), 950 µL supernatant were transferred to a new tube. The pellet was resuspended in 750 µL chloroform/methanol/37% (1N) HCl (40:80:1, *v*/*v*/*v*) and incubated for 15 min at room temperature with vortexing for 30 s every 5 min. Next, 250 µL cold chloroform and 450 µL cold 0.1 M HCl were added to the sample followed by vigorous vortexing for 1 min and centrifugation (6500× *g*, 2 min at 4 °C). The bottom organic phase was collected and pooled with the transferred supernatant from the first extraction. Subsequently, the sample was divided into two equal aliquots and dried using a SpeedVac concentrator. One aliquot was then dissolved in 50 µL solvent A/solvent B (2:1, *v*/*v*) for neutral and positive lipid analyses, and the other aliquot was reconstituted in 50 µL methanol for trimethylsilyldiazomethane (TMSD) methylation and anionic lipid analyses. For TMSD methylation, a solution of TMSD (2 mol/L) in hexane was added to the cellular lipid extracts to obtain yellow colored solutions. After vortexing for 30 s, methylation was performed at 37 °C for 15 min. Glacial acetic acid was added to quench the methylation reactions, yielding colorless samples. The samples were then subjected to LC–MS analysis.

### 2.13. Global Lipid Analysis Using LC–MS

Lipid analysis was performed using an Acquity ultra performance liquid chromatography (UPLC) system (Waters, Milford, MA, USA). A Hypersil GOLD column (2.1 × 100 mm inside diameter; 1.9 μm, Thermo Fisher Scientific) was used for separation of lipids. The temperatures of the column oven and the sample tray were set to 40 °C and 4 °C, respectively. Solvent A consisted of an acetonitrile:methanol:water mixture (19:19:2, *v*/*v*/*v*) with 20 mM ammonium formate and 0.1% (*v*/*v*) formic acid, and solvent B consisted of 2-propanol with 20 mM ammonium formate and 0.1% (*v*/*v*) formic acid. The gradient elution program was as follows: 0–5 min, B 5%; 5–15 min, B 5–30%; 15–22 min, B 30–90%; 22–25 min, B 90%; 25–26 min, 90–5%; and 26–30 min, B 5%. The flow rate was 250 μL/min, and the injection volume was 2 μL for each run. The total run time was 30 min. Lipids were analyzed using a QTRAP 5500 (AB Sciex) hybrid, triple quadrupole, linear ion trap MS instrument equipped with a Turbo V ion source, together with Analyst software, version 1.5.1. Ultra-high purity (UHP) nitrogen gas was used for the collisions. The following parameters were used with the operating source: 3500 V positive mode of capillary voltage, 3000 V negative mode of capillary voltage, a sheath gas flow rate of 11 L/min (UHP nitrogen) at 200 °C, drying gas flow rate of 15 L/min at 150 °C, and a nebulizer gas pressure of 25 psi. Optimized multiple reaction monitoring (MRM) conditions were used to analyze the various lipid species.

### 2.14. Processing Individual Data Points Obtained in MRM

LC–MS data were obtained and processed using Agilent Mass Hunter Workstation Data Acquisition software. The MRM data of target lipids, including the mass-to-charge ratios (*m*/*z*) of precursor ions, the *m*/*z* of product ions, and the retention time, were exported using Qualitative Analysis software, version B.06.00 (Agilent Technologies, Wilmington, DE, USA). Next, an in-house database constructed using Skyline software (MacCoss Laboratory, University of Washington, Seattle, WA, USA) was applied to determine the peak area of each assigned lipid, and all data were normalized by respective IS lipid. Statistical analyses such as PCA and hierarchical cluster analysis were performed using the MetaboAnalyst website. The volcano plot was generated by plotting statistical significance (*p*-value; −log10(*p*-value)) in the y-axis and magnitude of change between two groups (fold change; log2(fold change)) in the x-axis using Excel program.

### 2.15. Statistical Analysis

The data were analyzed using the Student’s *t*-test and expressed as the mean ± standard deviation. All experiments with primary human skin cells were independently performed at least three times, and representative results are shown. Lipidomic analyses on GrEVs and GrEXs were repeated nine times, respectively (n = 9 per group; three biological replicates, each with three technical replicates).

## 3. Results

### 3.1. Purification and Characterization of Extracellular Vesicles from Ginseng Roots

**Figure 1:**
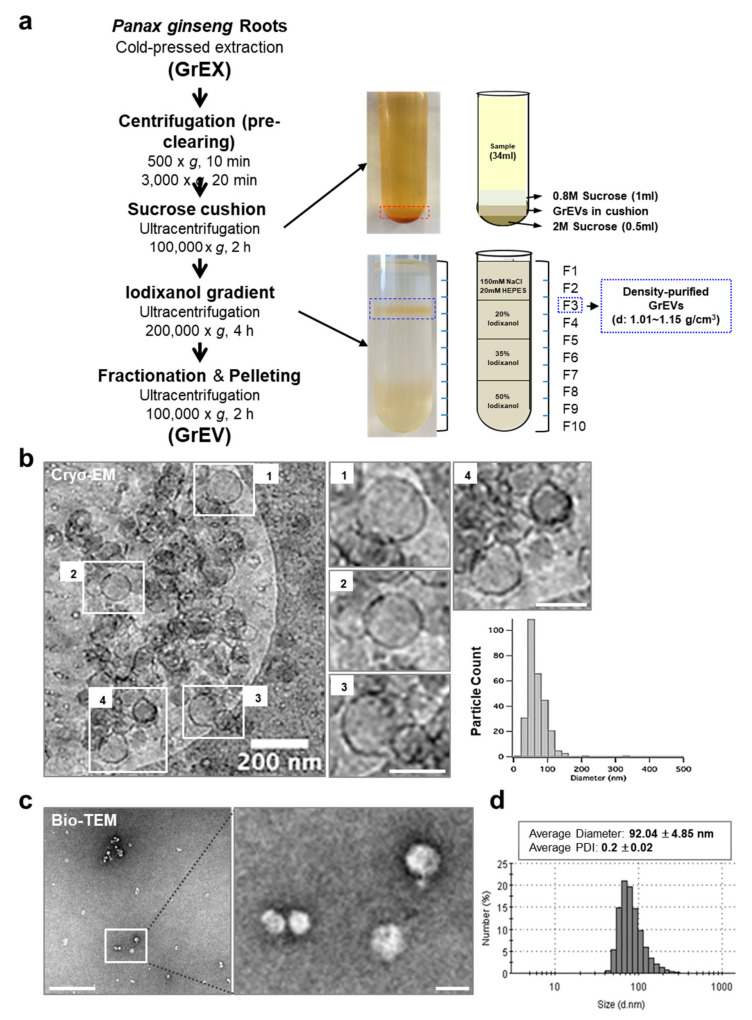
Purification and characterization of ginseng root-derived extracellular vesicles (GrEVs). (**a**) Purifying EVs from ginseng roots using sucrose cushion ultracentrifugation and density gradient ultracentrifugation. The GrEV fraction was located between the 0.8 M sucrose and the 2 M sucrose layers after sucrose cushion ultracentrifugation and was further purified using an iodixanol density gradient. Fraction 3 (F3; density purified GrEVs) with a density of 1.01–1.15 g/cm^3^ was collected and characterized biophysically. (**b**) Representative cryo-electron microscopy (cryo-EM) analysis of density purified GrEVs (left) and a size distribution based on individual cryo-EM images (right). Scale bars, 200 nm. (**c**) Representative bio-TEM images. Scale bars, 1 µm for low magnification; 100 nm for high magnification. TEM, transmission EM. (**d**) The size distribution of GrEVs was analyzed by dynamic light scattering. PDI, polydispersity index.

Ginseng root-derived EVs (GrEVs) were isolated from the cold-pressed ginseng root extracts (GrEXs) using a sucrose cushion and subsequent density gradient ultracentrifugation (Figure 1a). The GrEVs were located between 0.8 M and 2 M sucrose fractions after sucrose cushion ultracentrifugation, and this fraction was used to validate their biological effects in human skin cells. GrEVs were further fractionated by iodixanol gradient ultracentrifugation, after which GrEVs from fraction 3 (with a density of 1.01–1.15 g/cm^3^) were collected and used for morphological and lipidomic analyses, using cryo-electron microscopy (cryo-EM) and liquid chromatography–mass spectrometry (LC–MS), respectively. GrEVs revealed a closed spherical structure with diameter below 100 nm according to the cryo-EM and the bio-EM image analyses (Figure 1b,c) and showed an average diameter of 92.04 ± 4.85 nm and an average polydispersity index of 0.2 ± 0.02 based on dynamic light scattering (DLS) analysis (Figure 1d). These results suggest that the ginseng root cells release EVs of heterogeneous sizes, which can be isolated from ginseng extracts by a standard differential ultracentrifugation method.

### 3.2. GrEVs Reveal Anti-Senescence and Anti-Pigmentation Effects in Senescent Human Primary Cells

To examine the biological effects of GrEVs in human skin cells, primary keratinocytes were first cultured and treated with various doses of GrEVs for 4 days, with or without ultraviolet B (UVB) irradiation, and the expression levels of epidermal differentiation markers were determined. The expression levels of the early differentiation markers keratin 10 (*KRT10*) and terminal differentiation makers such as loricrin (*LOR*) and filaggrin (*FLG*) were not significantly changed by GrEV treatment under either normal or UV-irradiated conditions, although *LOR* and *FLG* expression seemed to be slightly upregulated by GrEV treatment (Figure S1a–c). However, secretion of the pro-inflammatory cytokine interleukin-6 (IL-6), which is associated with reduced terminal differentiation by *Staphylococcus aureus* or psoriasin and is induced by UV irradiation [46,47], was decreased by GrEV treatment under normal growth conditions (Figure S1d). These results suggest that GrEVs may in part contribute to recovery from ultraviolet radiation (UVR)-induced inflammation and disturbed terminal differentiation of the human epidermis.

**Figure 2:**
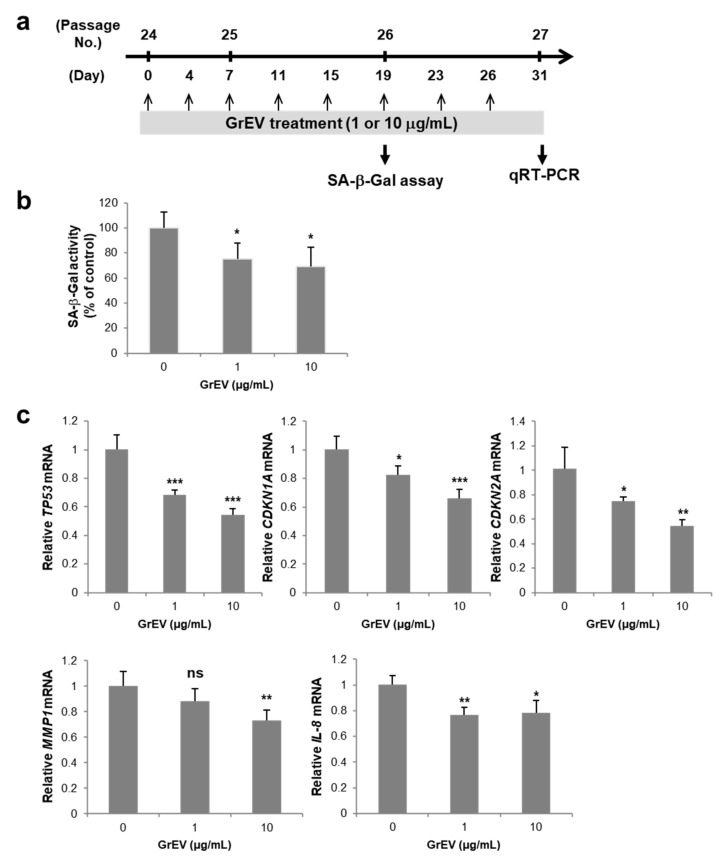
Ginseng root-derived extracellular vesicles (GrEVs) caused an anti-senescence effect in replicative senescent human dermal fibroblasts (HDFs). (**a**) Scheme used to treat replicative senescent HDFs with GrEVs. HDFs (2 × 10^5^), which were not treated (0 µg/mL; HEPES-buffered saline (HBS) buffer-treated) or were repeatedly treated with GrEVs at different doses (1 or 10 µg/mL) every third or fourth day, were harvested at passage numbers 26 and 27 (after > 50 population doublings). (**b**,**c**) The harvested cells were analyzed for senescence-associated beta-galactosidase (SA β-Gal) activity using a Mammalian β-Gal Assay Kit (**b**) and for gene expression by performing reverse transcriptase-quantitative polymerase chain reaction experiments using specific primers (**c**). SA β-Gal activity and gene expression levels were normalized to protein quantities and *RPL13A* expression, respectively. The data (in b and c) represent the means ± standard deviations of three independent experiments, using two independent senescent cell lines from one donor (* *p* < 0.05, ** *p* < 0.01, *** *p* < 0.001; ns, non-significant).

**Figure 3:**
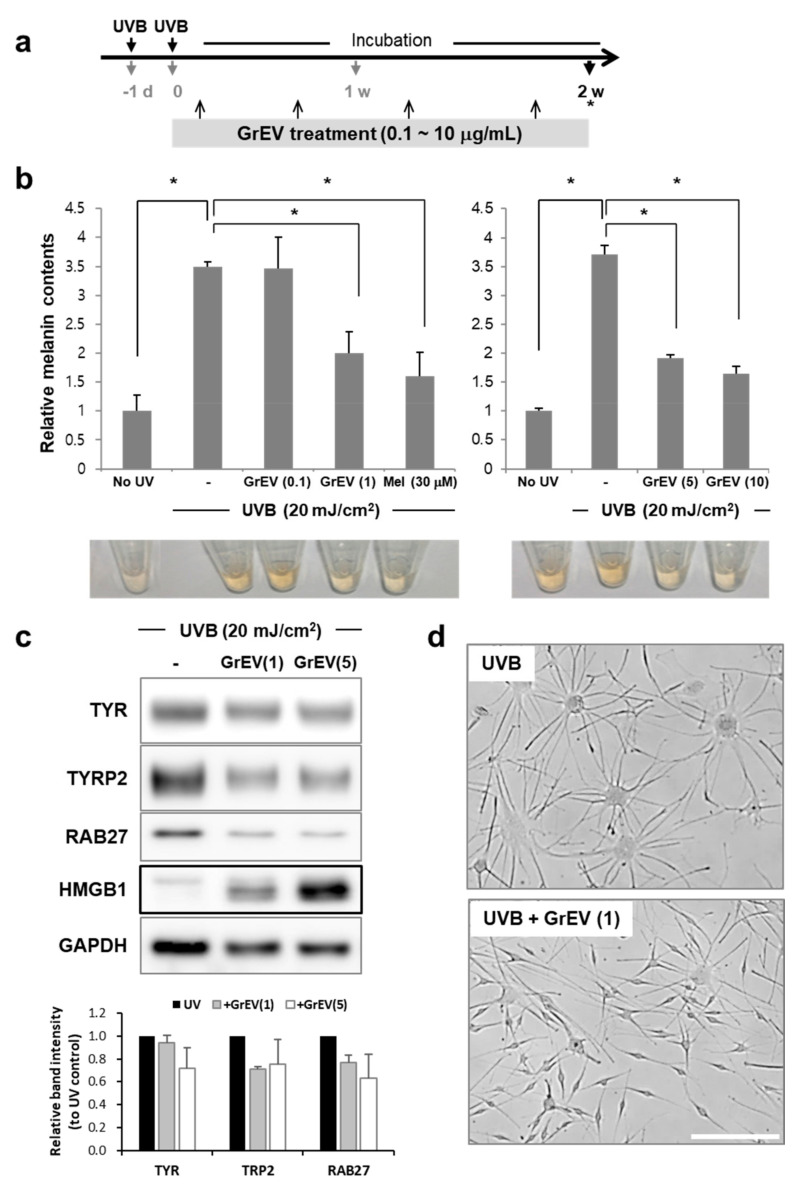
Ginseng root-derived extracellular vesicles (GrEVs) reveal anti-senescence and anti-pigmentation effects on human melanocytes with ultraviolet B (UVB) irradiation-induced senescence. (**a**) Scheme used to treat UVB-induced senescent human epidermal melanocytes (HEMs) with GrEVs. HEMs were exposed twice to UVB (20 mJ/cm^2^) over a 24 h interval and treated with GrEVs at different doses (0.1, 1, 5, or 10 µg/mL) or melasolv (30 µM) two times during a 2 week cultivation period. (**b**) Melanin contents were determined by measuring the absorbance at 450 nm after dissolving cell pellets in 1 N NaOH and normalizing to protein quantities. A representative image showing the color of the supernatant after dissolving in 1 N NaOH is shown in the bottom panel. The data are shown as the means ± standard deviations of three independent experiments (* *p* < 0.05). (**c**) Immunoblot analyses were performed on senescent HEMs treated with GrEVs (1 or 5 µg/mL) using the indicated antibodies. Twenty-five micrograms of protein were loaded, and glyceraldehyde 3-phosphate dehydrogenase (GAPDH) expression was detected as a loading control. Densitometry analysis of each band intensity was performed using ImageJ software (https://imagej.nih.gov/ij/). (**d**) Representative phase–contrast images of HEMs at 2 weeks post-treatment with GrEVs (0 or 1 µg/mL). Scale bar, 10 µm. (-) in b and c, HBS buffer-treated.

In contrast to the mild effect on epidermal differentiation, the anti-aging properties of ginseng and its active components have been well verified in human skin cells and in an animal model of aging [41]. To investigate whether GrEVs also elicit anti-aging effects, we induced replicative senescence in human dermal fibroblasts [48] and treated the cells with GrEVs on every third or fourth day over a 1 month period (Figure 2a), considering the need for long term treatment of anti-aging agents to restore the senescence phenotypes [9]. Senescence-associated (SA) β-galactosidase activity was significantly decreased by GrEV treatment in a dose-dependent manner (Figure 2b). Furthermore, the mRNA-expression levels of other SA markers such as p53 (*TP53*), p21^Cip1^ (*CDKN1A*), p16^INK4a^ (*CDKN2A*), matrix metalloprotease 1 (*MMP1*), and interleukin 8 (*IL-8*) were also gradually downregulated with increasing concentrations of GrEVs (Figure 2c). These results revealed that GrEVs overcame replicative senescence in fibroblasts when applied for a long period (1 month). As another model of senescent skin cells, we used melanocytes with UVB-induced senescence, which were established by exposing the cells twice to UVB in a 24 h period and cultivating them for 2 weeks (Figure 3a), during which time they developed pigmentation [42]. The melanin levels of UVB-induced senescent melanocytes were decreased significantly by various doses of GrEVs (1, 5, or 10 µg/mL) given over a 2 week period (Figure 3b) but not by a low concentration of GrEVs (0.1 µg/mL; Figure 3b). The effect of GrEVs on reducing melanin levels was comparable to that of a representative whitening substance, melasolv (3,4,5-trimethoxycinnamate thymol ester) [49,50], and of GrEXs at the same protein concentrations (Figure 3b and Figure S2a). Notably, treatment with a high concentration of GrEXs (10 µg/mL) seemed to be more cytotoxic to cells than GrEVs at the same concentration (Figure S2b). According to international organization for standardization (ISO) 10993-5, substances that do not decrease the viability below 80% are considered non-cytotoxic; therefore, GrEVs showing >80% viability at a high concentration (Figure S2b) appeared to be more effective and safer than GrEXs.

We examined the protein-expression levels of melanogenesis-related factors tyrosinase (TYR), tyrosinase-related protein 2 (TRP2), and Ras-related protein 27 (RAB27). The expression levels of these proteins following UVB irradiation appeared to be decreased by GrEV treatment in a dose-dependent manner (Figure 3c). In contrast, expression of the high mobility group box 1 (HMGB1) protein, which can inhibit UVB-induced apoptosis [51], was markedly increased by GrEVs in a dose-dependent manner (Figure 3c). Following these expression patterns of proteins, GrEV treatment reversed the UVB-induced senescent morphology of melanocytes (i.e., a flattened appearance and enlarged cell body, loss of fusiform, and high dendricity) (Figure 3d). These results suggest that GrEVs overcame replicative senescence in dermal fibroblasts and exerted anti-senescence effects on melanocytes with UVR-induced senescence, which was accompanied by an anti-pigmentation effect.

### 3.3. GcEVs Reveal Anti-Senescence and Anti-Pigmentation Effects in Senescent Human Primary Cells

**Figure 4:**
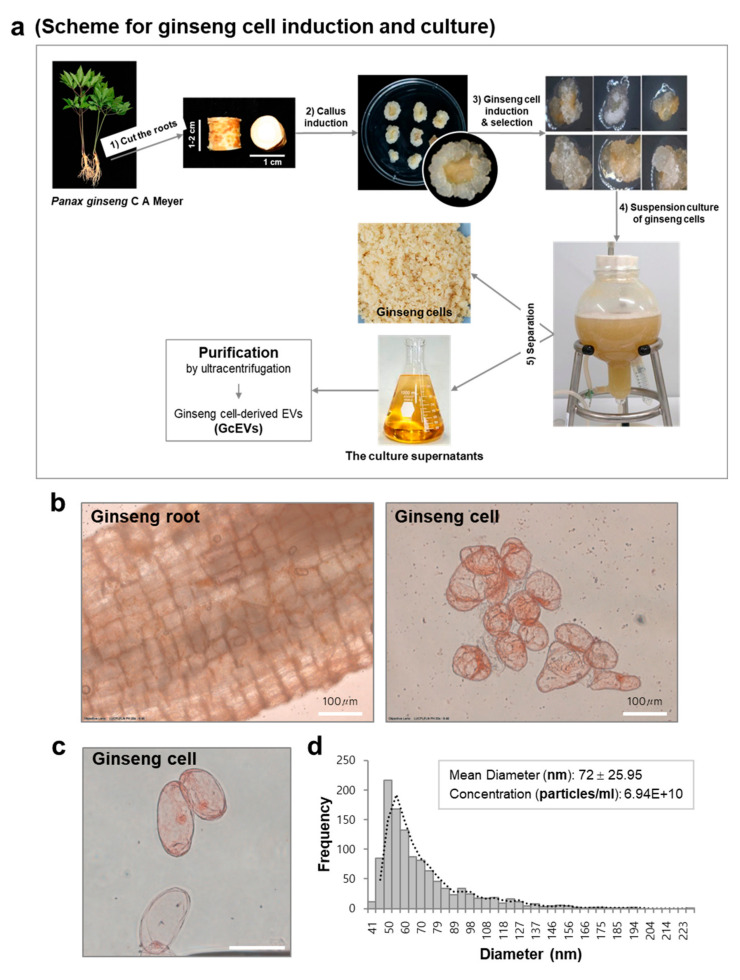
Isolation and characterization of ginseng cells and ginseng cell-derived EVs (GcEVs). (**a**) Diagrammatic representation of the process for isolating and culturing ginseng cells. After isolating ginseng cells from ginseng roots and culturing them in suspension for two weeks, GcEVs were purified from the culture supernatants by a conventional ultracentrifugation method. (**b**) Representative phase–contrast images of ginseng roots and ginseng cells. Scale bars, 100 µm. (**c**) Representative phase–contrast image of ginseng cells in culture. Scale bar, 100 µm. (**d**) The diameter and the particle number of GcEVs were analyzed by tunable resistive pulse sensing after ultracentrifugation.

In general, ginseng roots are harvested when the plants reach 4 to 6 years of age, peeled or steamed, and then dried to prepare white ginseng or red ginseng, respectively. Considering the time-consuming and the labor-intensive processes involved in long term field cultivation of ginseng roots, culturing cells and tissues from ginseng roots using a bioreactor represents an attractive alternative for producing ginseng biomass and active ingredients with a uniform quality and yield [43,52]. We investigated whether cultured ginseng cells could be used as a source of GEVs. Ginseng cells were derived from callus masses of *P. ginseng* root explants and grown on Murashige and Skoog medium supplemented with 2,4-dichlorophenoxyacetic acid (2,4-D), sucrose, and a gelling agent [43] (Figure 4a). Actively growing ginseng cell lines were further selected and cultured in bioreactors for 2 weeks in Marashige and Skoog media with 2,4-D (Figure 4a). After this process, the fresh weight (FW) of ginseng cells at 2 weeks post-inoculation (208 g/L) was approximately 7-fold higher than the initial FW (30 g/L), suggesting that the ginseng cell mass actively expanded in a short period.

Cultured ginseng cells showed relatively even spherical structures, ~50–100 µm in size range, with less-differentiated cell walls than observed in the ginseng roots (Figure 4b,c). Following a standard ultracentrifugation step, we purified EVs from the culture supernatants of ginseng cells, which are generally discarded without finding a use for them. Based on tunable resistive pulse sensing (TRPS) analysis, GcEVs had a mean diameter of 72 ± 25.95 nm, and the concentration was 6.94 × 10^10^ particles per ml (Figure 4d).

**Figure 5:**
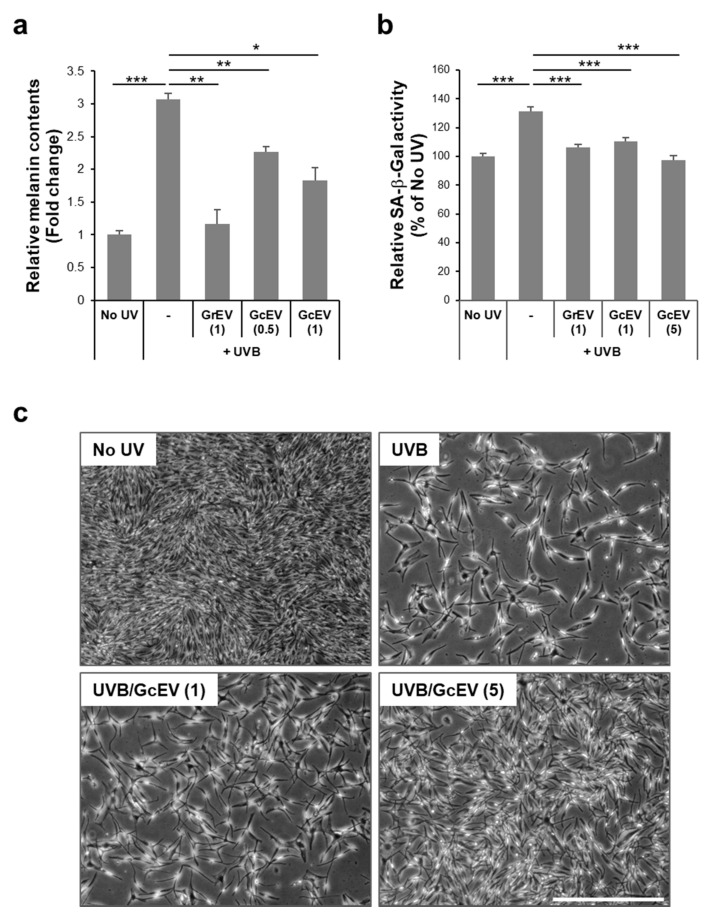
Ginseng cell-derived extracellular vesicles (GcEVs) showed anti-senescence and anti-pigmentation effects on melanocytes with ultraviolet B (UVB)-induced senescence. Human epidermal melanocytes (HEMs) were irradiated twice with UVB (20 mJ/cm^2^) over a 24 h interval and treated two times with ginseng root-derived extracellular vesicles (GrEVs; 1 µg/mL) or GcEVs at different doses (0.5, 1, or 5 µg/mL) during a 2 week cultivation period. (**a**) Melanin contents and (**b**) senescence-associated beta-galactosidase (SA β-Gal) activities were determined by measuring absorbance values at 450 nm after dissolving the cell pellets in 1 N NaOH or using a Mammalian β-gal Assay Kit, respectively. Melanin contents and SA β-Gal activities were normalized to protein quantities. The data are shown as the means ± standard deviations of three independent treatments (* *p* < 0.05, ** *p* < 0.01, *** *p* < 0.001). (**c**) Representative phase–contrast images of HEMs at 2 weeks post-treatment with GcEVs (1 or 5 µg/mL) under the condition of UVB-induced senescence. Scale bar, 50 µm. (-) in a and b, HBS buffer-treated.

We examined the cellular effects of GcEVs in UVB-induced senescent and pigmented melanocytes. GcEV treatment significantly and dose-dependently decreased the melanin contents of the senescent cells, and the effects were comparable to those caused by GrEV treatment (Figure 5a). Moreover, SA-beta-galactosidase activity was significantly and dose-dependently decreased by GcEVs to a greater extent than that observed after GrEV treatment (Figure 5b). UVB-induced senescent morphology of melanocytes, i.e., flattening and enlargement of the cell body, was rescued by GcEV treatment at two different doses, which was accompanied by an increased number of cells with a fusiform morphology (Figure 5c). These results suggest that cultured ginseng cells spontaneously released EVs possessing anti-senescence and anti-pigmentation activities against UVB-induced senescent melanocytes. Therefore, ginseng cells with less-differentiated cell walls and their culture supernatants can be potentially used for EV production and purification.

### 3.4. Differential Enrichment of Lipid Species between GEVs and Ginseng Root Extracts

**Figure 6:**
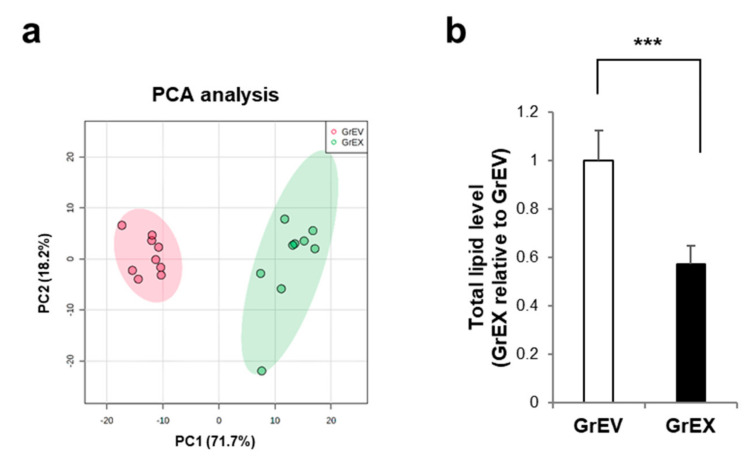
The discriminative lipidomic profile of ginseng root-derived extracellular vesicles (GrEVs). (**a**) Principal component analysis (PCA) of lipidomic information was performed based on the mass spectrometry (MS) data of GrEVs and ginseng root extracts (GrEXs). Lipidomic data were analyzed statically using the package in MetaboAnalyst (n = 9 per group; three biological replicates, each with three technical replicates). X axis: principal component 1 (PC1). Y axis: principal component 2 (PC2). (**b**) Relative total lipid expression levels in both groups. The expression levels of total lipids were determined by summing the amounts of identified lipid species in GrEVs and ginseng root extracts (GrEXs), respectively. The value of fold change is shown as the means ± standard deviations (n = 9 per group; three biological replicates, each with three technical replicates). Statistical significance was analyzed by Student’s *t*-tests for two groups (*** *p* < 0.001).

Edible plant-derived EVs have been investigated as delivery vehicles due to their biophysical characteristics, such as a lipid bilayer-enclosed, nanosized spherical structure, and their apparent safety. The lipid composition and the vesicular contents of plant-derived EVs differ from those of mammalian EVs; thus, they display differential delivery and vesicular functions with recipient cells [15]. To investigate whether the lipid composition of EVs differs from that of the parental ginseng root extracts, we performed a comprehensive lipidomic analysis of targeted lipids using multiple reaction monitoring (MRM) mode with a triple quadrupole MS instrument after separating the lipids by liquid chromatography (Figure S3). According to principal component analysis (PCA), which is performed to visualize the general clustering trends of two groups, the lipid profiles of GrEVs and GrEXs were distinct, showing 89.9% of variance contributed by 71.7% of principal component 1 (PC1) and 18.2% of principal component 2 (PC2) (Figure 6a).

A total of 188 lipid species, belonging to 15 different lipid classes, were identified from the GrEXs and GrEVs (n = 9 per group; three biological replicates, each with three technical replicates) based on their mass: charge (*m*/*z*) ratios and matching retention times, when compared to internal standards representing a specific lipid family (Table 1). We quantified the lipids identified in GrEVs and GrEXs by measuring the peak area of each lipid species relative to that of the corresponding internal standard. The expression intensities of the 188 lipid species between both groups are shown in a heat map (Figure S4a). Overall, GrEVs contained a 1.74-fold higher lipids:protein ratio than GrEXs (Figure 6b). The fold changes of the identified lipids between both groups are summarized by the class in Table 2. The lipid classes enriched in GrEVs included diacylglycerols (DG), phosphatidylcholine (PC), phosphatidylethanolamine (PE), lysophosphatidylcholine (LPC), and sphingomyelin (SM), all of which were significantly increased by more than two-fold in GrEVs when compared to GrEXs.

**Figure 7:**
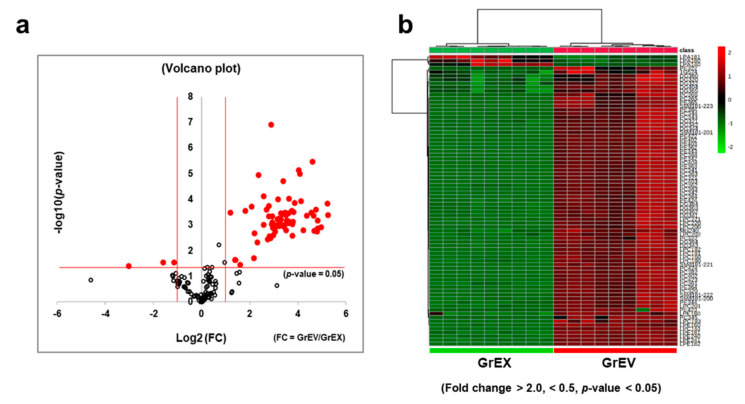
Differentially enriched lipid species and lipid composition and proportion in ginseng root-derived extracellular vesicles (GrEVs) versus ginseng root extracts (GrEXs). (**a**) The 188 identified lipids in GrEVs are represented in a volcano plot according to their statistical *p*-values and fold-changes when compared to those detected in GrEXs. The red lines at the left and the right sides represent distinct boundaries for lipid species with a <0.5-fold decrease or a >2.0-fold increase, respectively, in GrEVs (when compared to those in GrEXs. The horizontal red line represents the boundary for the significance (*p* < 0.05). (**b**) A heat map graphically represents the differential fold changes of 73 lipid species in GrEVs compared to the corresponding levels in GrEXs (fold change >2.0 or <0.5, *p* < 0.05). The relative differences are indicated by the color intensity, where red shading indicates increased levels and green shading indicates decreased levels.

A volcano plot depicting the distributions of the detected lipid species, based on the magnitudes and the significances of their differential signal intensities, reveals that many lipid species were significantly more abundant in GrEVs than in GrEXs (Figure 7a). In the plot, the vertical red lines represent distinct boundaries showing at least two-fold difference between GrEVs and GrEXs for increased or decreased lipids, respectively, and the horizontal red line represents a boundary for the significance (*p* = 0.05) (Figure 7a). Of the 188 identified lipids, 73 lipid species (38.8%) showed a statistically significant difference between the groups with 70 (95.9%) showing increase (fold change > 2; *p* < 0.05) and three (4.1%) showing decrease (fold change < 0.5; *p* < 0.05) in GrEVs compared to GrEXs (Figure 7a). These significantly and differentially fold changed lipid species are displayed on a heat map (Figure 7b), and the lists for differentially increased or decreased lipid species are described in Supplementary Tables S1 and S2, respectively, along with the fold changes and the statistical significances.

The largest number of differentially increased lipids in GrEVs was represented by PC (22) followed by PE (13), DG (11), LPC (11), LPE (6), SM (5), TG (1), and MG (1) (Table S1). The differentially decreased lipids were composed of LPA (3) (Table S2). MG (24:1), PC (42:2), PE (38:2), and SM (d18:1-22:0) were detected only in GrEVs, and six DG species including DG (34:1), DG (36:3), DG (34:2,) DG (36:4), DG (34:4), and DG (34:3), were ranked in the top ten together with LPC (18:2), LPE (24:0), PE (42:2), LPC (22:0), and LPC (18:1) (Table S1), suggesting that these species could be potential lipid biomarkers for GEVs. Taken together, these results suggest that, compared with the parent root extracts, GrEVs possess unique properties in terms of their lipid constitutions, which may be useful for evoking cellular responses or delivering bioactive constituents into the recipient human cells.

### 3.5. Comparison of the Lipid Composition in GEVs versus Ginseng Root Extracts

**Figure 8:**
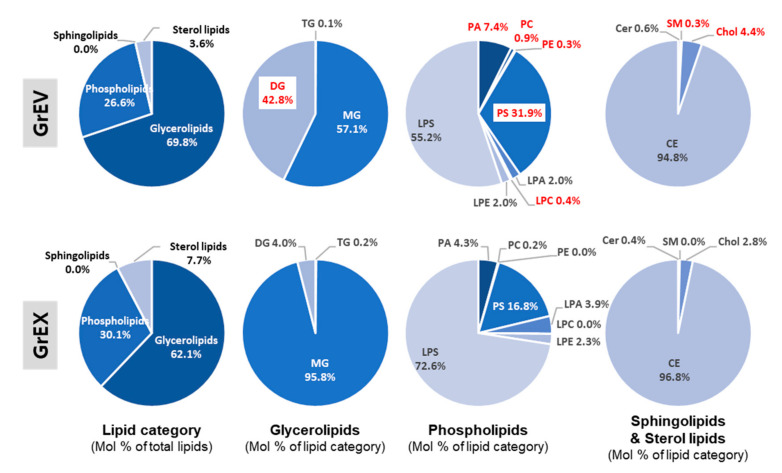
Pie diagrams for lipidomic compositions and relative proportions in GrEVs (upper) and GrEXs. The composition of each lipid category or lipid class (glycerolipids, phospholipids, and sphingolipids/sterol lipids) is expressed as the mol% of total lipids or the mol% of each lipid category. Lipid classes with considerable changes in GrEVs compared to GrEXs are indicated with red colored fonts; 100% by lipid class in GrEVs or GrEXs corresponds to the percentage of each lipid in lipid category.

We further examined compositional differences among the lipid classes in GrEVs and GrEXs by accessing the proportion of each lipid class with respect to the total lipids (mol% of total lipids). In particular, the proportions of DG and phospholipids (including PA, PC, PE, PS, and LPC) were higher in GrEVs, whereas those of MG, LPA, LPS, Chol, and CE were lower (Table 3). These findings suggest that GrEVs have vesicular characteristics with a lipid bilayer and their DG can serve as a potential signaling molecule. The lipid class that accounts for more than half in the total lipids were glycerolipids (including TG, MG, and DG) showing 69.8% and 62.1% of all lipids in GrEVs and GrEXs, respectively. Sterol lipids accounted for 3.6% and 7.7% and phospholipids (including eight classes) accounted for 26.6% and 30.1% of all lipids, respectively, but the proportion of sphingolipids including Chol and CE was overall very low in the groups (lipid category; Figure 8).

Assessing the mol% of each lipid class in the lipid category revealed that GrEVs contained similar ratios of MG (57.1%) and DG (42.8%), whereas GrEXs contained a relatively high ratio of MG (95.9%) (glycerolipids; Figure 8). Interestingly, DG was one of the lipid classes with the largest compositional change in two groups, showing an approximately 10.8-fold higher level in GrEVs (glycerolipids; Figure 8). The proportions of Chol, especially SM, which belongs to sterol lipid or sphingolipids, were increased in GrEVs (sphingolipids and sterol lipids; Figure 8). As expected from the increased abundances of lipid species in GrEVs (Figure 7; Table S1), there was a notable difference in compositions and proportions of the phospholipid classes. Among eight classes, the proportions of PA, PC, PE, PS, and LPC were increased in GrEVs compared to those in GrEXs, while those of LPA and LPS were decreased (phospholipids; Figure 8). Specifically, the abundances of PC, PE, and LPC were 5.1-, 8.7-, and 13.7-fold higher in GrEVs, respectively (phospholipids; Figure 8). These results suggest that GrEVs and their parental extracts had different compositions and proportions of lipid classes, especially with respect to DG, PC, PE, and LPC. These results may reflect compositional and structural characteristics of EVs, e.g., lipid bilayer-enclosed vesicles, and may be associated with the roles of EVs in delivering vesicular components to and evoking cellular responses in recipient human cells.

## 4. Discussion

As key mediators of cell-to-cell communications, the characteristics of EVs derived from mammalian or bacterial cells have been verified in terms of their structures, vesicular components, and pathophysiological functions. However, those of plant-derived EVs, especially in terms of the biological effects on human cells, are barely known. Here, we report for the first time that EVs derived from *P. ginseng* C.A. Meyer can rescue senescence-related phenotypes in human skin fibroblasts and melanocytes. We verified that the lipidomic profile of GEVs differs from that of the parental extracts based on increased proportions of DG and phospholipids (including PC, PE, and LPC), which suggests that the GEVs possessed unique characteristics.

Ginseng possesses multiple beneficial properties in terms of human health, and ginsenosides are the major constituents responsible for these activities [38,53]. However, large scale production of these phytochemicals requires the development of biotechnological or manufacturing processes in order to improve the efficacy and guarantee the quality [41,54]. Given that other constituents besides ginsenosides are also expected to exert pharmacological effects [38], further research on other types of constituents, manufacturing processes, and new biological activities of ginseng may be necessary to expand ginseng-related medicinal, pharmaceutical, or commercial applications. GEVs are prepared from ginseng root extracts or ginseng cell supernatants (without fermentation or organic acid pretreatment) and include potentially active biomolecules such as proteins, nucleic acids, metabolites, and lipids. Thus, GEVs can potentially serve as a novel source of bioactive materials for industrial applications, which would otherwise be discarded.

Plant-derived EVs have been considered as attractive vectors for delivering siRNAs, microRNAs, proteins, and therapeutic drugs in view of targeting delivery, safety, and the cost of large scale production [55,56]. EV lipids derived from plants can be further assembled as nanovectors for in vivo delivery using a standard method used for liposome fabrication [30,56]. Likewise, plant-derived EVs have received substantial attention as a potential means for drug delivery rather than their direct effects on human physiology. Although several reports showed that direct communication occurs between plant and mammalian cells via plant-derived EVs, the host cells studied were restricted to mouse intestinal cell types including stem cells, macrophages, and epithelial cells, or cancer cells with the objective of treatment [17,18,57,58,59]. Thus, the functional effects and mechanisms of action of plant-derived EVs on human tissues (particularly the skin and related cell types) are barely known. Recently, however, the immunomodulatory function of GEVs in mammalian immunity was reported [59]. GEVs induced macrophage polarization to the M1 phenotype via vesicular ceramides (Cers) and Toll-like receptor 4 on macrophages, thereby inhibiting melanoma growth [59].

In this study, we focused on the beneficial effects of GEVs (GrEVs and GcEVs) on human skin cells by investigating their effects in replicative senescent fibroblasts or UVB-induced senescent melanocytes [9,42]. In addition to their promising effects in terms of reversing age-related phenotypes, e.g., senescence and SA pigmentation, GEVs showed less cellular toxicity than GrEXs at a high concentration, suggesting that the GEVs can act as efficient nanocarriers for biological activity with lower toxicity than crude extracts or juice. Consistent with our results, low toxicity to recipient cells and good tissue targeting have been proposed as beneficial properties provided by plant derived EVs [56]. Therefore, GEVs can at least serve three functions (immunomodulation, anti-aging, and anti-pigmentation) by simultaneously enhancing cutaneous immunity and improving (intrinsic or extrinsic) senescent phenotypes, including SA pigmentation in human skin.

In this study, the vesicular constituents responsible for anti-senescence effects in human skin cells were not verified. However, we found that HMGB1 protein levels markedly increased after GEV treatment in melanocytes with UVB-induced senescence. HMGB1 is a redox-sensitive and multifunctional protein with both harmful and beneficial activities, which is functionally regulated in different ways depending on its location in the nucleus, the cytoplasm, or the extracellular milieu [60,61]. In the nucleus, HMGB1 maintains the nuclear structure and regulates gene transcription; however, HMGB1 can be released into the extracellular space under conditions of stress (including pathological conditions such as autoimmune diseases) where it acts as a damage associated molecular protein (DAMP) that triggers innate immune responses by binding to the receptor for advanced glycation end products (RAGE) or toll-like receptors (TLRs) on surrounding cells [60,61,62,63,64,65]. In human skin, HMGB1 actively migrates from the nucleus to the extracellular milieu with age or under senescence conditions, for example, in p16^INK4A^-positive senescent melanocytes in older subjects, and is associated with senescence phenotypes such as the increased cytokine levels [66,67]. Therefore, we speculate that the elevated HMGB1 levels in cell lysates (including both the nucleus and the cytosol) after GEV treatment may represent cellular recovery from senescent phenotypes, e.g., the recovery of proliferation and pro-autophagic activity. Both of these processes were previously verified to be associated with high HMGB1 expression in the cytosol of young or undifferentiated cells [68,69].

Given that GEVs play immunomodulatory roles by inducing M1 macrophage polarization, which can be also mediated by HMGB1 [59,70], it is possible that, in GEV-treated melanocytes, HMGB1 expression may be increased intracellularly via DG-dependent protein kinase C (PKC) signaling and possibly released in an EV-associated form as opposed to a soluble form. Vesicular HMGB1 may participate in GEV-induced M1 macrophage polarization (resulting in increased skin immunity) in collaboration with other vesicular constituents such as ceramide. Therefore, important topics of further research include determining (i) which constituents of GEVs are involved in increasing HMGB1 protein expression in human melanocytes, (ii) which cellular functions are mediated by HMGB1 upregulation, and (iii) whether EVs derived from GEV-treated melanocytes harbor the HMGB1 protein.

Based on a comprehensive lipidomic analysis of GEVs and the parental extracts, DG, some phospholipids (PC, PE, and LPC), and SM were enriched by over 20-, 8-, and 7-fold in GrEVs, respectively, compared to the corresponding levels in GrEXs (Figure 6b). In general, the lipid DG functions as a secondary messenger by activating the PKC- and the protein kinase D (PKD) signaling pathways, thereby regulating cell cycle progression, cellular survival, malignant transformation, apoptosis, and melanogenesis [71,72,73]. In cancer cells, DG differentially accumulated in EVs according to the malignancy of the cells, and EVs enriched with DG activated the PKC signaling pathway in surrounding cells but not in the EV producing cells [74]. Therefore, besides the proposed role in lymphocyte membrane invagination [75,76], DG associated with GEVs may evoke responses in human skin cells by activating PKC signaling, thereby mediating inter-cellular communication between plant and human cells. Given that PKC can in turn regulate HMGB1 activity via phosphorylation [77], the increased intracellular expression of HMGB1 in GEV-treated melanocytes may be partially attributable to vesicular DG-activated PKC signaling. Thus, it may be worth investigating whether GEVs can activate PKC signaling and whether inhibition of that signaling can eliminate GEV-induced anti-senescence phenotypes in human melanocytes.

## 5. Conclusions

In this study, we verified the spontaneous release of EVs from ginseng cells in ginseng roots and cultured cells as well as their functional effects on the recovery from cellular senescence in human primary skin cells. Most active constituents in ginseng are extracted using organic solvents, and the remaining constituents (including active biomolecules such as proteins and lipids) and culture supernatants are discarded. Therefore, GEVs represent a potent alternative source of bioactive substances derived from ginseng for food products and topical applications. Based on our lipidomic analysis of GEVs, we suggest that DG is a potential GEV lipid biomarker that can mediate intercellular communication and directly evoke cellular responses in human cells via PKC signaling.

## Figures and Tables

**Table 1:** The classes of lipids in GrEVs and GrEXs detected by liquid chromatography–mass spectrometry. The number of identified lipid species in each lipid class was shown in descending order.

Lipid Class	Lipid Species Quantitated
Triacylglycerol (TG)	40
Phophatidylcholine (PC)	23
Lysophosphatidylethanolamine (LPE)	22
Phosphatidylethanolamine (PE)	19
Diacylglycerol (DG)	19
Cholesterol Ester (CE)	15
Lysophosphatidylcholine (LPC)	11
Monoacylglycerol (MG)	9
Lysophosphatidic acid (LPA)	9
Sphingomyeline (SM)	6
Phosphoserine (PS)	5
Lysophosphoserine (LPS)	4
Phosphatidic acid (PA)	3
Ceramide (Cer)	2
Cholesterol (Chol)	1
**TOTAL**	188

**Table 2:** Summary of the fold changes of lipid classes in GrEVs relative to those in GrEXs. The fold change values are shown as the means ± standard deviations (n = 9 per group; three biological replicates, each with three technical replicates). Statistical significance was analyzed by Student’s *t*-tests for two groups.

Lipid Category	Lipid Class	Fold Change ^(a)^	*p*-Value
		(Mean ± S.D.)	
Glycerolipids	MG	1.174 ± 0.051	6.06 × 10^−2^
	DG	21.470 ± 1.758	1.31 × 10^−3^
	TG	1.131 ± 0.099	4.95 × 10^−1^
Glycero- phospholipids	PA	2.507 ± 0.806	3.83 × 10^−1^
	PC	8.105 ± 1.042	1.00 × 10^−3^
	PE	12.293 ± 1.514	5.96 × 10^−4^
	PS	2.978 ± 0.187	6.93 × 10^−2^
	LPA	0.777 ± 0.158	3.44 × 10^−1^
	LPC	17.692 ± 0.530	9.31 × 10^−5^
	LPE	1.350 ± 0.202	5.52 × 10^−2^
	LPS	1.287 ± 0.381	6.16 × 10^−1^
Sphingolipids	Cer	1.084 ± 0.007	5.86 × 10^−2^
	SM	7.656 ± 0.448	6.74 × 10^−4^
Sterol lipids	Chol	1.461 ± 0.517	5.52 × 10^−1^
	CE	0.884 ± 0.340	6.67 × 10^−1^

^(a)^ GrEV/GrEX.

**Table 3:** The compositional proportions of lipid classes in GrEVs versus GrEXs. Each mol% represents the proportion of each lipid class relative to the total lipid contents, which was defined as the sum of the amount of identified lipid species determined by measuring its peak area relative to that of the corresponding internal standard (n = 9 per group; three biological replicates, each with three technical replicates). Lipid classes that increased in GrEVs or GrEXs are indicated by light or dark gray colors, respectively.

Lipid Category	Lipid Class	GrEV (Mol %)	GrEX (Mol %)
Glycerolipids	TG	0.07	0.11
	MG	39.85	59.54
	DG	29.90	2.47
Glycero-phospholipids	PA	1.96	1.30
	PC	0.23	0.05
	PE	0.07	0.01
	PS	8.48	5.05
	LPA	0.53	1.17
	LPC	0.11	0.01
	LPE	0.53	0.68
	LPS	14.66	21.86
Sphingolipids	Cer	0.02	0.03
	SM	0.01	0.00
Sterol lipids	Chol	0.16	0.22
	CE	3.43	7.50
	Sum	100	100

## Data Availability

Not applicable.

## Supplementary Materials

The following are available online at https://www.mdpi.com/2073-4409/10/3/486/s1, Figure S1: Effects of ginseng root-derived extracellular vesicles (GrEVs) in human epidermal keratinocytes, Figure S2: Comparison of the anti-melanogenic and cytotoxic effects of ginseng root-derived extracellular vesicles (GrEVs) and ginseng root extracts (GrEXs) in human epidermal melanocytes (HEMs), Figure S3: Workflow of lipidomic analysis for ginseng root-derived extracellular vesicles (GrEVs) and ginseng root extracts (GrEXs), Figure S4: Hierarchical clustering of all identified lipid species in ginseng root-derived extracellular vesicles (GrEVs) versus ginseng root extracts (GrEXs), Table S1: The list of differentially increased lipid species in ginseng root-derived extracellular vesicles (GrEVs) compared to in ginseng root extracts (GrEXs), Table S2: The list of differentially decreased lipid species in ginseng root-derived extracellular vesicles (GrEVs) compared to in ginseng root extracts (GrEXs).
