# Supplementary material for: Panax ginseng-Derived Extracellular Vesicles Facilitate Anti-Senescence Effects in Human Skin Cells: An Eco-Friendly and Sustainable Way to Use Ginseng Substances
Source: Cells. 2021 Feb 24;10(3):486. doi: 10.3390/cells10030486 (PMC7996359; doi:10.3390/cells10030486)
Supplement: Supplementary file 1 [file cells-10-00486-s001.pdf]

Supplementary information

Panax ginseng-derived extracellular vesicles facilitate anti-senescence effects in human skin cells: an eco-friendly and sustainable way to use ginseng substances

Eun-Gyung Cho, Suh-Yeon Choi, Hyoseon Kim, Eun-Jeong Choi, Eun-Jeong Lee, Phil-Jun Park, Jaeyoung Ko, Kwang Pyo Kim, and Heung Soo Baek

Supplementary Figures

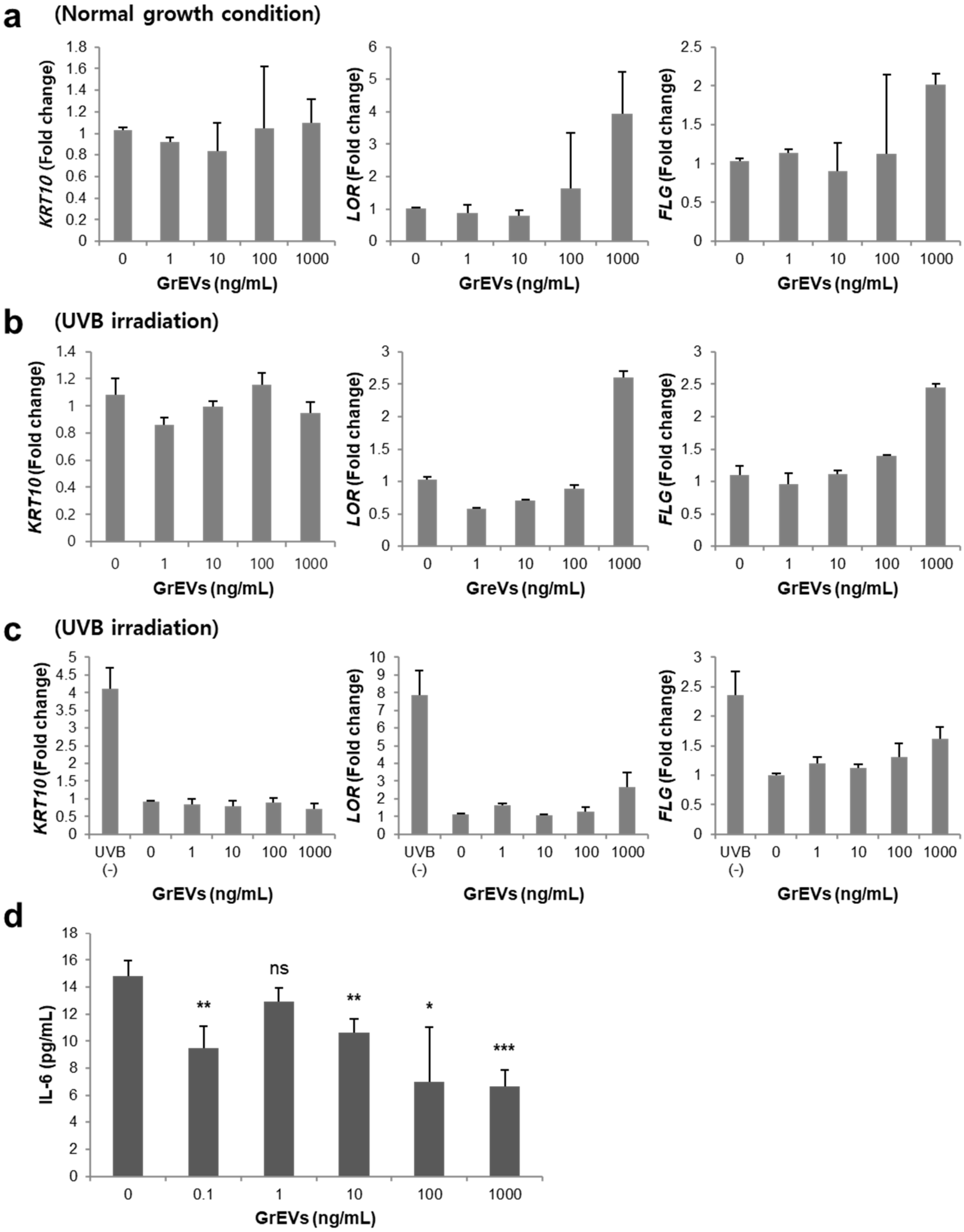

Figure S1 Effects of ginseng root-derived extracellular vesicles (GrEVs) in human epidermal keratinocytes. (a) Human epidermal keratinocytes (HEKs) were treated with various

concentrations (0, 0.1, 1, 10, 100, or 1000 ng/mL) of GrEVs and incubated for 12 days. (b, c) HEKs were irradiated with ultraviolet B (UVB; 30 mJ/cm^2), treated with the indicated concentrations of GrEVs, and incubated for 4 days. The gene-expression levels of epidermal differentiation markers (*KRT10*, *LOR*, and *FLG*) were assessed by reverse transcriptase-quantitative polymerase chain reaction analysis, using specific primers, and normalized to those of *RPL13A*. (d) HEKs were treated with indicated concentrations of GrEVs and incubated for 4 days. Secreted levels of the IL-6 protein were determined using an enzyme-linked immunosorbent assay kit for IL-6. The data shown in panels a–d represent the means ± standard deviations of three independent treatments (*$p < 0.05$, **$p < 0.01$, ***$p < 0.001$; ns, non-significant).

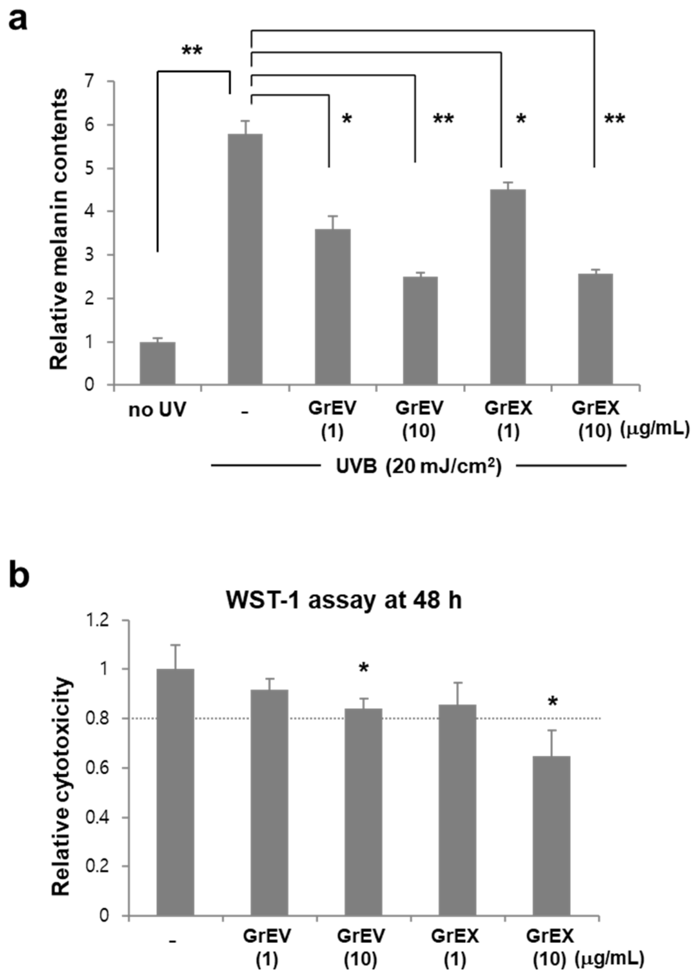

Figure S2 Comparison of the anti-melanogenic and cytotoxic effects of ginseng root-derived extracellular vesicles (GrEVs) and ginseng root extracts (GrEXs) in human epidermal melanocytes (HEMs). (a) HEMs were exposed twice to ultraviolet B (UVB) irradiation (20 mJ/cm^2) over a 24-h period and treated two times with different doses (1 or 10 μg/mL) of GrEVs or GrEXs during a 2-week cultivation period. Melanin contents were determined by measuring absorbance values at 450 nm after dissolving cell pellets in 1 N NaOH. Melanin levels were normalized to protein quantities in each sample. (b) HEMs (7×10^3) were treated with different doses (1 or 10 μg/mL) of GrEVs or GrEXs, and cell viabilities were assessed after 48 h using a cell-proliferation reagent (WST-1). The data shown in panels a and b represent the means ± standard deviations of three independent treatments (*$p < 0.05$, **$p < 0.01$).

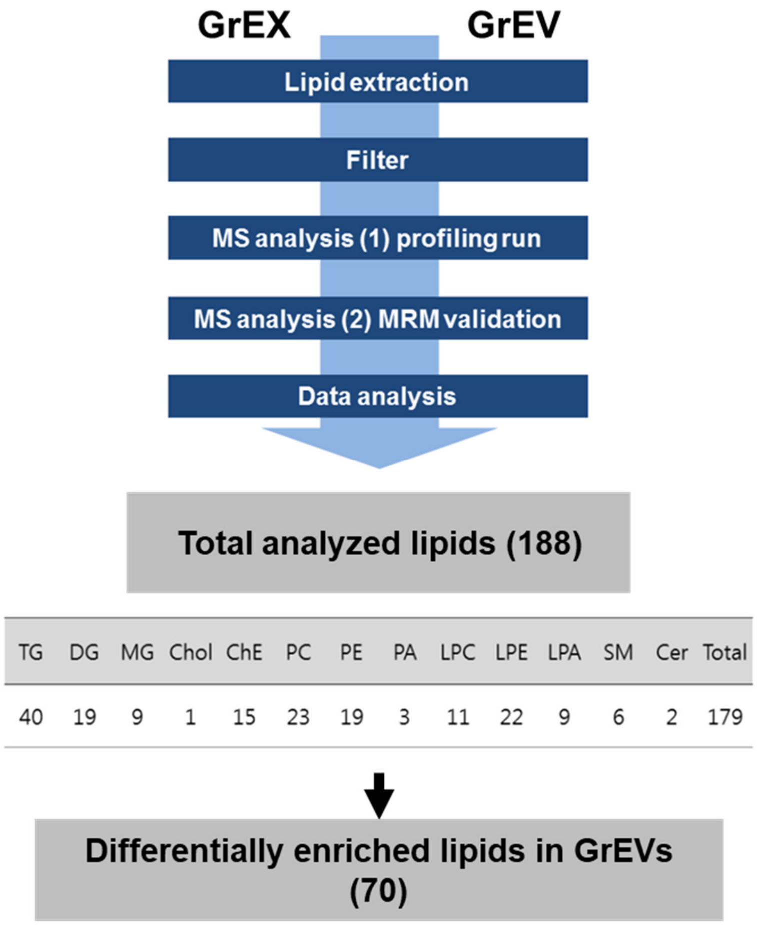

TG	DG	MG	Chol	ChE	PC	PE	PA	LPC	LPE	LPA	SM	Cer	Total
40	19	9	1	15	23	19	3	11	22	9	6	2	179

Figure S3 Workflow of lipidomic analysis for ginseng root-derived extracellular vesicles (GrEVs) and ginseng root extracts (GrEXs). The procedure used for liquid chromatography–mass spectrometry analysis is described in detail in the Experimental procedures section. We identified 188 different lipid species, among which 70 were differentially accumulated in GrEVs when compared to GrEXs.

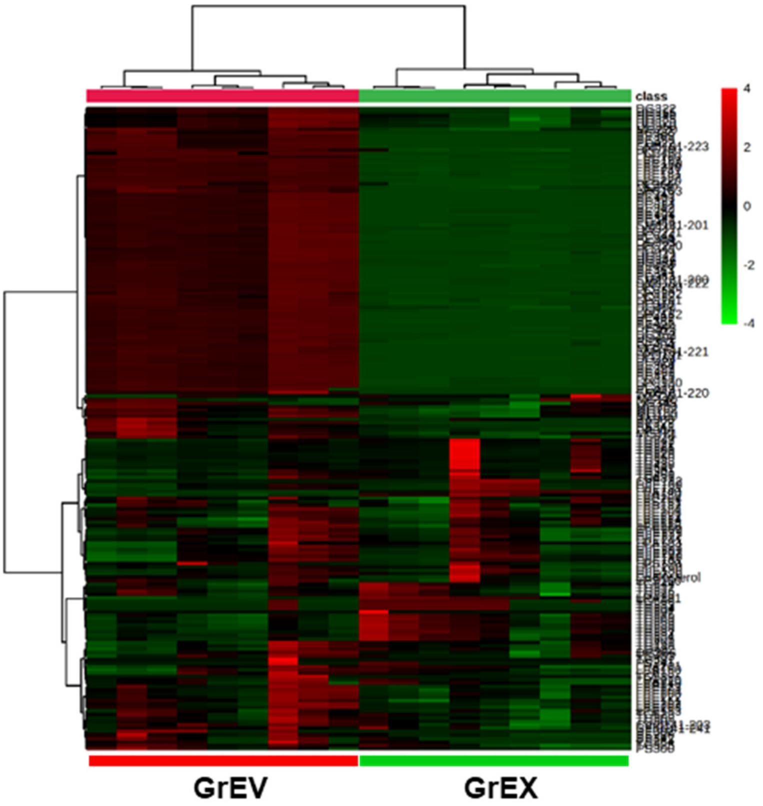

Figure S4 Hierarchical clustering of all identified lipid species in ginseng root-derived extracellular vesicles (GrEVs) versus ginseng root extracts (GrEXs). A heat map of 188 identified lipid species graphically represents the levels of differentially accumulated lipids between GrEVs and GrEXs. The relative differences are indicated by the color intensity, where red shading indicates increased levels and green shading indicates decreased levels.

No.	Lipids	Fold change[a)]	S.D.[b)]	*p*-value[c)]
1	**MG(24:1)**	n.d in GrEX	n.d	5.08E-05
2	**PC(42:2)**	n.d in GrEX	n.d	3.39E-04
3	**PE(38:2)**	n.d in GrEX	n.d	1.32E-02
4	**SM(d18:1-22:0)**	n.d in GrEX	n.d	6.43E-04
5	**LPC(18:2)**	**37.28**	3.14	1.52E-04
6	**DG(34:1)**	**31.22**	10.12	1.24E-03
7	**DG(36:3)**	**28.04**	4.14	1.40E-03
8	**DG(34:2)**	**27.77**	0.98	1.83E-03
9	**DG(36:4)**	**26.70**	2.42	2.57E-04
10	**DG(34:4)**	**25.48**	8.68	1.77E-03
11	**DG(34:3)**	**25.05**	2.37	4.52E-04
12	**LPE(24:0)**	**24.49**	0.66	3.46E-06
13	**PE(42:2)**	**23.71**	0.98	3.43E-04
14	**LPC(22:0)**	**18.79**	1.96	1.62E-03
15	**LPC(18:1)**	**17.42**	1.01	1.22E-04
16	**LPE(24:1)**	**16.81**	6.27	1.01E-05
17	**LPC(16:1)**	**16.27**	0.65	7.44E-06
18	**PE(40:2)**	**14.68**	0.73	1.43E-04
19	**PC(40:2)**	**14.15**	0.97	3.14E-04
20	**PE(36:5)**	**13.81**	2.15	7.60E-04
21	**LPC(20:0)**	**13.72**	1.05	9.65E-04
22	**DG(32:1)**	**13.66**	2.62	2.63E-03
23	**PC(42:3)**	**13.49**	0.93	2.83E-04
24	**PE(42:3)**	**12.47**	1.09	9.48E-05
25	**PE(34:2)**	**12.28**	1.78	8.38E-04
26	**SM(d18:1-22:3)**	**12.17**	1.60	7.25E-04
27	**PE(40:3)**	**12.16**	1.11	3.60E-04
28	**PE(34:3)**	**11.63**	1.41	8.60E-04
29	**PE(36:3)**	**11.62**	1.65	6.91E-04
30	**PE(36:4)**	**11.48**	1.25	4.26E-04
31	**LPC(22:1)**	**11.47**	1.79	8.91E-04
32	**PC(36:5)**	**11.45**	2.21	1.60E-03
33	**PE(36:2)**	**10.99**	0.97	3.39E-04
34	**PC(42:4)**	**10.94**	0.74	3.83E-04

35	**PC(38:4)**	**10.67**	1.86	1.19E-03
36	**LPC(16:0)**	**10.48**	0.72	1.96E-05
37	**PC(38:2)**	**10.40**	1.09	3.82E-04
38	**PC(32:1)**	**9.92**	1.41	1.00E-03
39	**LPC(20:2)**	**9.76**	0.53	3.53E-04
40	**PC(40:3)**	**9.76**	1.18	6.15E-04
41	**PC(40:4)**	**9.43**	1.40	8.14E-04
42	**PC(34:1)**	**9.36**	0.85	1.09E-03
43	**PC(36:3)**	**9.25**	1.26	1.00E-03
44	**PE(34:4)**	**9.21**	0.87	5.68E-04
45	**LPC(18:3)**	**9.00**	1.07	1.03E-04
46	**PC(36:1)**	**8.94**	1.73	6.03E-04
47	**PC(38:3)**	**8.82**	0.85	1.09E-03
48	**PC(36:2)**	**8.51**	0.58	1.05E-03
49	**PE(38:4)**	**8.29**	1.45	1.78E-03
50	**PC(34:3)**	**7.95**	1.42	2.48E-03
51	**PC(36:4)**	**7.78**	1.20	1.30E-03
52	**LPC(20:1)**	**7.70**	0.17	4.57E-04
53	**SM(d18:1-22:2)**	**7.69**	0.58	8.06E-04
54	**PC(34:2)**	**7.61**	0.97	9.69E-04
55	**SM(d18:1-20:1)**	**7.50**	0.69	9.29E-04
56	**LPE(18:2)**	**7.44**	0.33	1.28E-07
57	**PC(36:6)**	**7.29**	1.69	3.23E-03
58	**PC(34:4)**	**7.06**	1.35	2.74E-03
59	**SM(d18:1-20:0)**	**6.97**	0.37	4.59E-04
60	**PC(32:0)**	**6.90**	0.44	1.87E-04
61	**PC(34:5)**	**6.72**	1.67	3.79E-03
62	**SM(d18:1-22:1)**	**6.49**	0.34	2.58E-04
63	**LPE(16:0)**	**5.99**	0.24	7.78E-05
64	**PC(32:2)**	**5.98**	0.75	9.78E-04
65	**LPE(18:1)**	**5.12**	0.27	1.16E-05
66	**DG(36:5)**	**5.02**	1.52	4.77E-03
67	**DG(32:2)**	**4.62**	0.50	2.17E-03
68	**DG(36:0)**	**4.49**	1.10	1.95E-02
69	**LPC(18:0)**	4.24	0.08	1.97E-04

70	**LPE(18:0)**	**3.53**	0.83	2.87E-04
71	**PE(32:2)**	**3.01**	1.03	3.63E-02
72	**TG(52:4)**	**2.67**	0.57	2.38E-02
73	**DG(32:0)**	**2.63**	0.25	2.33E-02
74	**MG(24:0)**	**2.28**	0.06	3.42E-04

[a)] GrEV/GrEX > 2; [b)] Standard Deviation; [c)] p-value < 0.05

Table S1 The list of differentially increased lipid species in ginseng root-derived extracellular vesicles (GrEVs) compared to in ginseng root extracts (GrEXs). The data are shown as the means ± standard deviations of the fold-increases with the corresponding *p*-values (n = 9 per group; three biological replicates, each with three technical replicates). The list is displayed in descending order of fold change. Statistical significance was analyzed by Student's *t*-tests for two groups.

No.	Lipids	Fold change[a)]	S.D.[b)]	*p*-value[c)]
1	**LPA(14:0)**	**0.12**	0.04	4.15E-02
2	**LPA(18:1)**	**0.33**	0.02	2.99E-02
3	**LPA(18:0)**	**0.45**	0.10	2.97E-02

[a)] GrEV/GrEX < 0.5; [b)] Standard Deviation; [c)] *p*-value < 0.05

Table S2 The list of differentially decreased lipid species in ginseng root-derived extracellular vesicles (GrEVs) compared to in ginseng root extracts (GrEXs). The data are shown as the means ± standard deviations of the fold-decrease with the corresponding *p*-values (n = 9 per group; three biological replicates, each with three technical replicates). The list is displayed in descending order of fold change. Statistical significance was analyzed by Student's *t*-tests for two groups.
